# Supplementary material for: The Role of the NRF2 Pathway in the Pathogenesis of Viral Respiratory Infections
Source: Pathogens. 2023 Dec 31;13(1):39. doi: 10.3390/pathogens13010039 (PMC10819673; doi:10.3390/pathogens13010039)
Supplement: Supplementary file 1 [file pathogens-13-00039-s001.zip › pathogens-2783251-supplementary.pdf]

Supplementary Table S1: Impact of non-respiratory viruses on the Nrf2 pathway

Viruses	Mechanism of Nrf2 activation	Reference
HIV gp120 and Tat proteins, NS3 viral protein of HCV, other HCV viral proteins, Marburg virus (MARV) VP24 protein	**Viral proteins:**<br>• ↑ direct activation of Nrf2<br>• HCV NS3 binds to cytosolic sMafs that regulate Nrf2.<br>• HCV viral proteins ↑ ROS through a Ca2+ signaling in the ER and ↑ Nrf2/ARE signaling.<br>• MARV VP24 binds and ↓ Keap1 ↑ Nrf2	[1-7]
HPV	Hypermethylation of the KEAP1 gene promoter CpG island ↓ Keap1 ↑ Nrf2	[8]
Herpes viruses such as KSHV and EBV	• Herpes viruses ↓ Bach1, a major Keap1-independent regulator of Nrf2 ↑ Nrf2	[9,10]
DNA viruses that can induce latency such as KSHV, HBV and RNA viruses such as SFTS	• Alter autophagy, ↑ P62 ↓ KEAP1 binding to Nrf2 ↑ Nrf2	[11-16]
HCV	• ↑ phosphorylation of the redox-sensitive PKC ↑ NRF2 dissociation from KEAP1	[17]
Hepatitis viruses	• ↑ PI3K-protein kinase B (Akt) signaling pathway which ↑ phosphorylation of GSK3 and cytosolic NRF2 to further ↑ its proteasomal degradation through a CULLIN1 (CUL1)-dependent mechanism	[18,19]
Dengue virus	• ↑ Nrf2 through the ER stress-protein kinase RNA-like ER kinase (PERK) signaling pathway	[20]
Human cytomegalovirus (HCMV)	• ↑ ROS-independent casein kinase 2 (CK2) pathway ↑ Nrf2	[21]
Coxsackievirus B3 (CVB3)	• ↑ 12/15-lipoxygenase (12/15-LO) (a lipid-peroxidizing enzyme involved in production of inflammatory mediators) ↓ Nrf2 in cardiac myocytes	[22]
DNA viruses that can induce latency such as KSHV	• The viral genome can directly associate with Nrf2 in a manner that is dependent on the latency-associated KSHV protein LANA-1, which allows for the transcription of the latent viral proteins	[23]

Abbreviations: ROS: Reactive oxygen species; mito-ROS: mitochondrial Reactive oxygen species, ER: endoplasmic reticulum, sMafs: small Maf proteins, Nrf2: Nuclear factor erythroid 2-related factor, HCV, Hepatitis C virus, KEAP1: Kelch-like-ECH-associated protein 1, RSV: Respiratory syncytial virus, KSHV: Kaposi sarcoma-associated herpesvirus, EBV: Epstein Barr Virus, HBV: Hepatitis B virus, SFTS: Severe fever with thrombocytopenia syndrome, PKC: protein kinase C, PI3K: phosphatidylinositol 3-kinase (PI3K), GSK3: glycogen synthase kinase 3, COX-2: cyclooxygenase 2. PGE2: Prostaglandin E2.

References in Supplemental Material

1. Staitieh, B.S.; Ding, L.; Neveu, W.A.; Spearman, P.; Guidot, D.M.; Fan, X. HIV-1 decreases Nrf2/ARE activity and phagocytic function in alveolar macrophages. *J Leukoc Biol* **2017**, *102*, 517-525, doi:10.1189/jlb.4A0616-282RR.
2. Mastrantonio, R.; Cervelli, M.; Pietropaoli, S.; Mariottini, P.; Colasanti, M.; Persichini, T. HIV-Tat Induces the Nrf2/ARE Pathway through NMDA Receptor-Elicited Spermine Oxidase Activation in Human Neuroblastoma Cells. *PLoS One* **2016**, *11*, e0149802, doi:10.1371/journal.pone.0149802.
3. Carvajal-Yepes, M.; Himmelsbach, K.; Schaedler, S.; Ploen, D.; Krause, J.; Ludwig, L.; Weiss, T.; Klingel, K.; Hildt, E. Hepatitis C virus impairs the induction of cytoprotective Nrf2 target genes by delocalization of small Maf proteins. *J Biol Chem* **2011**, *286*, 8941-8951, doi:10.1074/jbc.M110.186684.
4. Vomund, S.; Schafer, A.; Parnham, M.J.; Brune, B.; von Knethen, A. Nrf2, the Master Regulator of Anti-Oxidative Responses. *Int J Mol Sci* **2017**, *18*, doi:10.3390/ijms18122772.
5. Ivanov, A.V.; Smirnova, O.A.; Ivanova, O.N.; Masalova, O.V.; Kochetkov, S.N.; Isaguliants, M.G. Hepatitis C virus proteins activate NRF2/ARE pathway by distinct ROS-dependent and independent mechanisms in HUH7 cells. *PLoS One* **2011**, *6*, e24957, doi:10.1371/journal.pone.0024957.
6. Edwards, M.R.; Johnson, B.; Mire, C.E.; Xu, W.; Shabman, R.S.; Speller, L.N.; Leung, D.W.; Geisbert, T.W.; Amarasinghe, G.K.; Basler, C.F. The Marburg virus VP24 protein interacts with Keap1 to activate the cytoprotective antioxidant response pathway. *Cell Rep* **2014**, *6*, 1017-1025, doi:10.1016/j.celrep.2014.01.043.
7. Page, A.; Volchkova, V.A.; Reid, S.P.; Mateo, M.; Bagnaud-Baule, A.; Nemirov, K.; Shurtleff, A.C.; Lawrence, P.; Reynard, O.; Ottmann, M.; et al. Marburgvirus hijacks nrf2-dependent pathway by targeting nrf2-negative regulator keap1. *Cell Rep* **2014**, *6*, 1026-1036, doi:10.1016/j.celrep.2014.02.027.
8. Ma, J.Q.; Tuersun, H.; Jiao, S.J.; Zheng, J.H.; Xiao, J.B.; Hasim, A. Functional Role of NRF2 in Cervical Carcinogenesis. *PLoS One* **2015**, *10*, e0133876, doi:10.1371/journal.pone.0133876.

9. Komaravelli, N.; Ansar, M.; Garofalo, R.P.; Casola, A. Respiratory syncytial virus induces NRF2 degradation through a promyelocytic leukemia protein - ring finger protein 4 dependent pathway. *Free Radic Biol Med* **2017**, *113*, 494-504, doi:10.1016/j.freeradbiomed.2017.10.380.

10. Yin, Q.; McBride, J.; Fewell, C.; Lacey, M.; Wang, X.; Lin, Z.; Cameron, J.; Flemington, E.K. MicroRNA-155 is an Epstein-Barr virus-induced gene that modulates Epstein-Barr virus-regulated gene expression pathways. *J Virol* **2008**, *82*, 5295-5306, doi:10.1128/JVI.02380-07.

11. Jiang, T.; Harder, B.; Rojo de la Vega, M.; Wong, P.K.; Chapman, E.; Zhang, D.D. p62 links autophagy and Nrf2 signaling. *Free Radic Biol Med* **2015**, *88*, 199-204, doi:10.1016/j.freeradbiomed.2015.06.014.

12. Komatsu, M.; Kurokawa, H.; Waguri, S.; Taguchi, K.; Kobayashi, A.; Ichimura, Y.; Sou, Y.S.; Ueno, I.; Sakamoto, A.; Tong, K.I.; et al. The selective autophagy substrate p62 activates the stress responsive transcription factor Nrf2 through inactivation of Keap1. *Nat Cell Biol* **2010**, *12*, 213-223, doi:10.1038/ncb2021.

13. Gjyshi, O.; Flaherty, S.; Veettil, M.V.; Johnson, K.E.; Chandran, B.; Bottero, V. Kaposi's sarcoma-associated herpesvirus induces Nrf2 activation in latently infected endothelial cells through SQSTM1 phosphorylation and interaction with polyubiquitinated Keap1. *J Virol* **2015**, *89*, 2268-2286, doi:10.1128/JVI.02742-14.

14. Gjyshi, O.; Roy, A.; Dutta, S.; Veettil, M.V.; Dutta, D.; Chandran, B. Activated Nrf2 Interacts with Kaposi's Sarcoma-Associated Herpesvirus Latency Protein LANA-1 and Host Protein KAP1 To Mediate Global Lytic Gene Repression. *J Virol* **2015**, *89*, 7874-7892, doi:10.1128/JVI.00895-15.

15. Liu, B.; Fang, M.; He, Z.; Cui, D.; Jia, S.; Lin, X.; Xu, X.; Zhou, T.; Liu, W. Hepatitis B virus stimulates G6PD expression through HBx-mediated Nrf2 activation. *Cell Death Dis* **2015**, *6*, e1980, doi:10.1038/cddis.2015.322.

16. Choi, Y.; Jiang, Z.; Shin, W.J.; Jung, J.U. Severe Fever with Thrombocytopenia Syndrome Virus NSs Interacts with TRIM21 To Activate the p62-Keap1-Nrf2 Pathway. *J Virol* **2020**, *94*, doi:10.1128/JVI.01684-19.

17. Smirnova, O.A.; Ivanova, O.N.; Mukhtarov, F.S.; Tunitskaya, V.L.; Jansons, J.; Isaguliants, M.G.; Kochetkov, S.N.; Ivanov, A.V. Analysis of the Domains of Hepatitis C Virus Core and NS5A Proteins that Activate the Nrf2/ARE Cascade. *Acta Naturae* **2016**, *8*, 123-127.

18. Guo, H.; Zhou, T.; Jiang, D.; Cuconati, A.; Xiao, G.H.; Block, T.M.; Guo, J.T. Regulation of hepatitis B virus replication by the phosphatidylinositol 3-kinase-akt signal transduction pathway. *J Virol* **2007**, *81*, 10072-10080, doi:10.1128/JVI.00541-07.

19. Rada, P.; Rojo, A.I.; Chowdhry, S.; McMahon, M.; Hayes, J.D.; Cuadrado, A. SCF/beta-TrCP promotes glycogen synthase kinase 3-dependent degradation of the Nrf2 transcription factor in a Keap1-independent manner. *Mol Cell Biol* **2011**, *31*, 1121-1133, doi:10.1128/MCB.01204-10.

20. Cheng, Y.L.; Lin, Y.S.; Chen, C.L.; Tsai, T.T.; Tsai, C.C.; Wu, Y.W.; Ou, Y.D.; Chu, Y.Y.; Wang, J.M.; Yu, C.Y.; et al. Activation of Nrf2 by the dengue virus causes an increase in CLEC5A, which enhances TNF-alpha production by mononuclear phagocytes. *Sci Rep* **2016**, *6*, 32000, doi:10.1038/srep32000.

21. Lee, J.; Koh, K.; Kim, Y.E.; Ahn, J.H.; Kim, S. Upregulation of Nrf2 expression by human cytomegalovirus infection protects host cells from oxidative stress. *J Gen Virol* **2013**, *94*, 1658-1668, doi:10.1099/vir.0.052142-0.

22. Ai, F.; Zheng, J.; Zhang, Y.; Fan, T. Inhibition of 12/15-LO ameliorates CVB3-induced myocarditis by activating Nrf2. *Chem Biol Interact* **2017**, *272*, 65-71, doi:10.1016/j.cbi.2017.05.010.

23. Gjyshi, O.; Bottero, V.; Veettil, M.V.; Dutta, S.; Singh, V.V.; Chikoti, L.; Chandran, B. Kaposi's sarcoma-associated herpesvirus induces Nrf2 during de novo infection of endothelial cells to create a microenvironment conducive to infection. *PLoS Pathog* **2014**, *10*, e1004460, doi:10.1371/journal.ppat.1004460.
